# Structural and Immunological Activity Characterization of a Polysaccharide Isolated from *Meretrix meretrix* Linnaeus

**DOI:** 10.3390/md14010006

**Published:** 2015-12-29

**Authors:** Li Li, Heng Li, Jianying Qian, Yongfeng He, Jialin Zheng, Zhenming Lu, Zhenghong Xu, Jinsong Shi

**Affiliations:** School of Pharmaceutical Science, Jiangnan University, Wuxi 214122, China; li792051095@163.com (L.L.); eternal83@163.com (H.L.); jackieqian@163.com (J.Q.); 15061888901@163.com (Y.H.); 18262280354@163.com (J.Z.); zhenming_lu@163.com (Z.L.); zhenghxu@jiangnan.edu.cn (Z.X.)

**Keywords:** *Meretrix meretrix* Linnaeus, polysaccharide, structure, immunological activity

## Abstract

Polysaccharides from marine clams perform various biological activities, whereas information on structure is scarce. Here, a water-soluble polysaccharide MMPX-B2 was isolated from *Meretrix meretrix* Linnaeus. The proposed structure was deduced through characterization and its immunological activity was investigated. MMPX-B2 consisted of d-glucose and d-galctose residues at a molar ratio of 3.51:1.00. The average molecular weight of MMPX-B2 was 510 kDa. This polysaccharide possessed a main chain of (1→4)-linked-α-d-glucopyranosyl residues, partially substituted at the C-6 position by a few terminal β-d-galactose residues or branched chains consisting of (1→3)-linked β-d-galactose residues. Preliminary immunological tests *in vitro* showed that MMPX-B2 could stimulate the murine macrophages to release various cytokines, and the structure-activity relationship was then established. The present study demonstrated the potential immunological activity of MMPX-B2, and provided references for studying the active ingredients in *M. meretrix*.

## 1. Introduction

*Meretrix meretrix* Linnaeus (*M. meretrix*, Meretrix, Veneridae) is a historically marine food and a valuable source of traditional Chinese medicine (TCM), which is widely distributed in coastal areas of South and Southeast Asia [1]. Documented in the ancient Chinese pharmacopeia *Compendium of material* (the 16th century, by Li Shizhen), *M. meretrix* could diminish inflammation, treat typhoid fever, hangover and relieve pain. Modern research has verified the effects of *M. meretrix* on immuno-regulating [2], antioxidant [3], antineoplastic [4], antihypertensive and hypolipidemic activities [5]. The noteworthy biological activities relied on the functional ingredients it contains, including peptides, polysaccharides, amino acids, and enzyme inhibitors. Among these compounds, polysaccharide is one of the major components which accounted for 4.1%–8.3% of *M. meretrix* [2]. Various biological functions of polysaccharides including immunomodulation, anti-inflammation, anti-coagulation, and anti-tumor have been explored and evaluated [6]. A series of immunological indicators, including the phagocytic power, the number of leukocytes, and the level of hemolysin antibody, were all ameliorated in the rats with damaged immune systems induced by cyclophosphamide through the oral administration of a polysaccharide isolated from *M. meretrix* [2]. Zhang *et al.* also reported that the ethanol extract of *M. meretrix* could enhance the expression of T- and B- lymphocytes by 18% and 43%, respectively [7]. The underlying mechanism through which polysaccharides exerted their physiological activities has experienced a long exploring process, but a rapid advancement has been achieved recently, mainly because of the discovery of roles of gut microbiota. Polysaccharides which could not be absorbed into the small intestine could be further hydrolyzed and metabolized in the colon, which might exert some influences on the physiological features and community structure of gut microbiota [8,9]. Chang *et al.* showed comprehensively that the high molecular weight polysaccharides (4300 kDa) isolated from *Ganoderma lucidum* (a medicinal mushroom) mycelium reduced body weight, inflammation and insulin resistance in mice fed a high-fat diet (HFD) by reversing HFD-induced gut dysbiosis, maintaining the intestinal barrier integrity, and reducing metabolic endotoxemia [10].

The progress in the mechanism elucidation further forced the interests in the research of novel polysaccharides in clams and also in their structure-activity relationship. A polysaccharide extracted from *Cyclina sinensis* performed high inhibitory activity *in vitro* against human gastric cancer cells. It was composed of glucose linked by α-(1→4) glycosidic bonds, with branches attached to the backbone chain by (1→6) glycosidic bonds [11]. Liao *et al.* also isolated a polysaccharide from the clam of *Corbicula fluminea* with significant inhibitory effects on growth of human gastric cancer cells and human ovarian carcinoma cells [12]. In addition, Vidhyanandhini *et al.* obtained purified glycosaminoglycans from *Meretrix casta*, whose structural characterization was carried out by Fourier transform-infrared (FT-IR) and ^1^H-NMR spectroscopy [13]. These glycosaminoglycans showed comparable anticoagulant activity with heparin [14]. However, the current research on polysaccharides of *M. meretrix* is still confined to the extraction method improvement and primary bio-activity evaluation. Detailed characterization of structure and elucidation of structure-activity relationship are still scarce.

In the present study, a polysaccharide was isolated from *M. meretrix* and purified. The structure was deduced and proposed based on detailed chemical characterization, and the immuno-regulatory activity was evaluated. An attempt to find a preliminary structure-activity relationship was carried out as well.

## 2. Results and Discussion

### 2.1. Extraction and Purification of MMPX-B2

**Figure 1:**
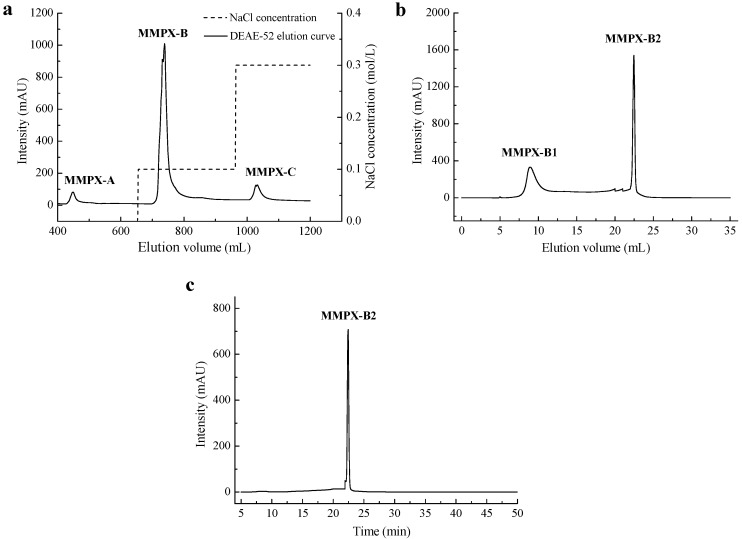
Purification of MMPX-B2. (**a**) Elution profile of crude polysaccharides by DEAE-52 cellulose; (**b**) Purification profile of MMPX-B by Superdex 200; (**c**) HP-GPC profile of MMPX-B2.

The crude polysaccharide (MMPX) was extracted from *M. meretrix* following an enzymatic extraction method with addition of 2% trypsin. Under the optimized conditions, the yield of MMPX reached 12.0%. As preliminary evaluation showed, this crude polysaccharide could increase NO production in RAW264.7 cell; however, a higher purity and homogeneity of the polysaccharide was needed in order to analyze its structure and bio-activity analysis. Therefore, MMPX was then isolated by DEAE-52 cellulose anion-exchange chromatography. The chromatogram (Figure 1a) showed three peaks, in which the major part eluted by 0.1 M NaCl was collected and denoted as MMPX-B. MMPX-B was further purified by Superdex 200 dextran gel permeation chromatography, and two fractions were isolated. As shown in Figure 1b, two peaks arose, and the constituent which performed a symmetrical and sharper peak was collected for subsequent studies and denoted as MMPX-B2. The purity and homogeneity of MMPX-B2 were further investigated. There was no absorption at 260 nm and 280 nm (data not shown) in UV spectrum, indicating no impurities of protein and nucleic acids left. The high performance gel permeation chromatography (HP-GPC) profile in Figure 1c showed only one symmetrical peak, indicating that MMPX-B2 was a homogenous polysaccharide, with an apparent molecular weight of 510 kDa. Furthermore, the polydispersity index (PDI) was determined as 1.11 corresponding to a narrow molecular weight distribution [15], suggesting a relative simple composition of MMPX-B2.

### 2.2. Chemical Composition of MMPX-B2

**Figure 2:**
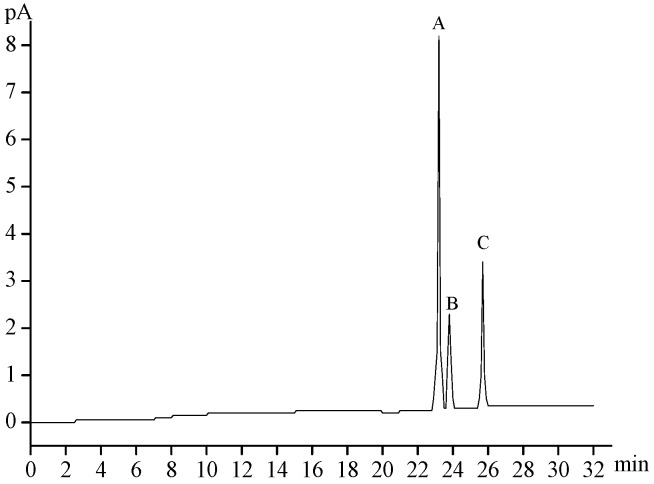
GC profile of MMPX-B2 with acid hydrolysis and acetylation. (**A**) d-glucose; (**B**) d-galactose; (**C**) internal standard inositol.

A gas chromatography (GC) analysis (Figure 2) showed that MMPX-B2 was composed of d-Glucose and d-Galactose with the molar ratio of 7.13:1.00, indicating that this polysaccharide was neutral.

**Figure 3:**
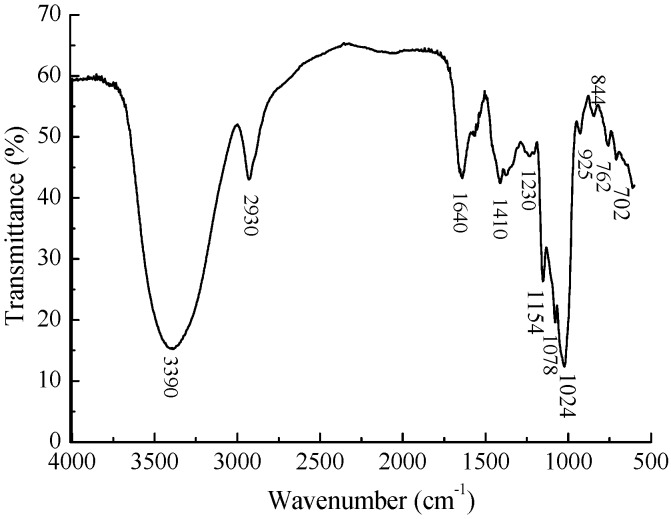
FT-IR spectrum of MMPX-B2.

The structure was further analyzed by FT-IR. As shown in Figure 3, a broad and intense characteristic peak at 3390 cm^−1^ was attributed to the stretching vibration of O–H. The bands at 2930 cm^−1^ were assigned to C–H_2_ and C–H stretching vibrations [16]. The relatively strong absorption at 1640 cm^−1^ was due to associated water [17]. Additionally, 1410 cm^−1^ and 1078 cm^−1^ corresponded to exocyclic and endocyclic C–O stretching bands, respectively [18]. The peaks around 1400–1200 cm^−1^ were also the characteristic absorptions of C–H bonds. The absorptions at 1200–950 cm^−1^ were due to the vibrations of C–O–H side groups and C–O–C glycosidic bonds [16]. The band around 900 cm^−1^ was the characteristic absorption of β-linkage of pyranose [19,20]. The relatively intensive bonds at 700–900 cm^−1^ were assigned to skeletal modes of a pyranose ring, among which the absorption at 844 cm^−1^ suggested the presence of α-type glycosidic bonds [21].

### 2.3. Linkage Analysis and Structure Speculation

Preliminary analysis of the glycosidic linkage locations was carried out by following periodate oxidation and Smith degradation methods.

The results of periodate oxidation showed that, per mole sugar, 0.960 mol of periodate was consumed and 0.112 mol formic acid was produced. The formation of formic acid suggested the presence of pyranohexose in 1→ or 1→6 linked forms in 11.2%. As the amount of periodate consumption was more than two fold of the amount of formic acid produced, linkages which only consumed periodate without formic acid production were therefore deduced to exist as 1→2, 1→2,6, 1→4, and 1→4,6 forms, which occupied 73.6% of the total glycosyl linkages. The ratio of other linkages as 1→3-linked forms were 15.2% which did not consume periodate.

Additionally, large amounts of glycerol and erythritol, examined by gas chromatography-mass spectrometry (GC-MS) after the periodate-oxidized products of MMPX-B2, were reduced and hydrolyzed indicating that most of the linkages were in 1→, 1→6, 1→2, 1→2,6, 1→4, and 1→4,6 forms. Combined with the results of periodate oxidation analysis, it could be inferred that linkages of 1→2, 1→2,6, 1→4, 1→4,6 were the major types. Nevertheless, small amounts of 1→3 glycosyl linkages also exist.

Methylation analysis was employed for further derivation of glycosyl linkages in MMPX-B2. The results (Table 1) showed that the glucose residues were linked together as (1→4)-Glc*p* and (1→4,6)-Glc*p* forms, while the galactose residues were present as terminal and (1→3)-linked Gal*p* forms. Further, the ratio of four types of glycosyl linkages were determined by integrating peak areas. The number of (1→4)-linked and (1→4,6)-linked Glc*p* residues accounted for 77.22% of the total methylated sugar residues, suggesting that MMPX-B2 was probably consisted of a backbone of 1,4-linked and 1,4,6-linked Glc*p*, with terminal Gal*p* residues (majority) and branches of 1,3-linked Gal*p* (minority) attached to the C-6 of some 1,4,6-linked Glc*p* residues. There is also some possibility of a few 1,3-linked Gal*p* residues inside the Glc*p* main chain. Further structural data are needed for structure speculation.

**Table 1:** GC-MS analysis of the methylated products of MMPX-B2.

Methylated Sugar Residue	Molar Ratio	Ratio (%)	Type of Linkage
2,3,4,6-Me_4_-Gal	1.03	17.64	Gal*p*-(1→
2,4,6-Me_3_-Gal	0.30	5.14	→3)-Gal*p*-(1→
2,3,6-Me_3_-Glc	3.51	60.10	→4)-Glc*p*-(1→
2,3-Me_2_-Glc	1.00	17.12	→4,6)-Glc*p*-(1→

**Figure 4:**
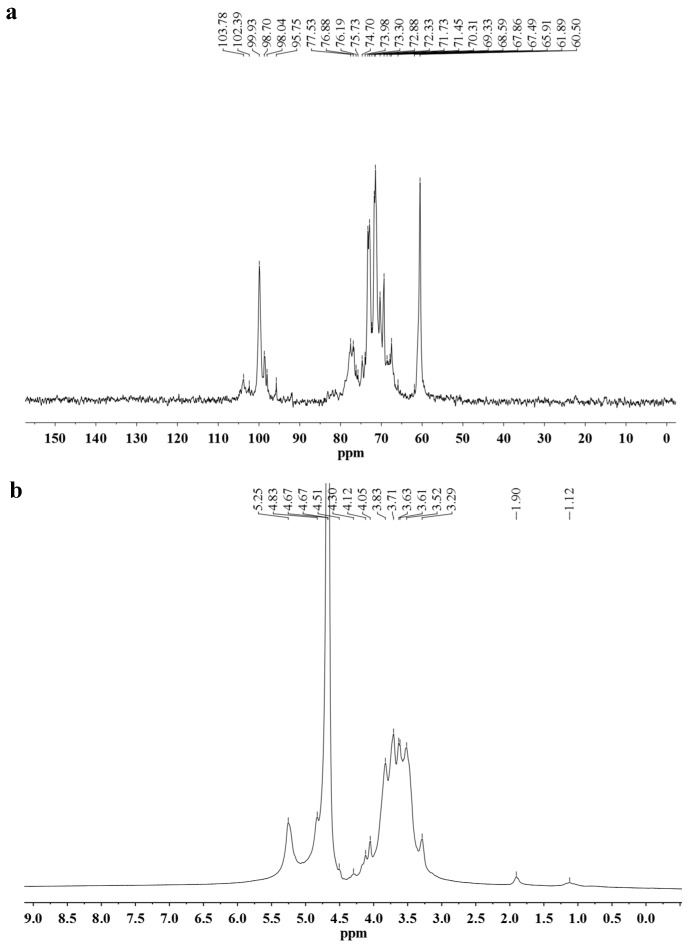
NMR spectra of MMPX-B2. (**a**) ^13^C-NMR; (**b**) ^1^H-NMR.

As the MMPX-B2 structure deduced was not complicated, 1D NMR spectroscopy was chosen to provide a more exact structural information (Figure 4). The major chemical shifts are listed in Table 2, and the assignments were mainly based on literature values. As MMPX-B2 was composed almost exclusively by glucose, the signals of glucose and those of galactose could be easily differentiated. In the ^13^C NMR spectrum, the resonances in the anomeric region (δ = 95–110 ppm) allowed for quick assignments of the configurations of the sugar residues, as α-configuration at δ = 95–102 ppm and β-configuration at δ = 103–110 ppm [22]. The signals at δ 98.70 and 99.92 ppm were attributed to the anomeric carbon atoms of (1→4,6)-linked α-d-Glc*p* and (1→4)-linked α-d-Glc*p* [23,24], while the signals at δ 103.78 and 102.39 ppm were ascribed to the terminal β-d-Gal*p* and (1→3)-linked β-d-Gal*p* [25]. The signal in the lower magnetic field at δ 60.50 ppm was attributed to C-6 resonance of →4)-α-d-Glc*p*-(1→. In addition, ^1^H-NMR spectrum showed intensive and broad peaks in the range of 3.0–4.5 ppm, which gave additional information for structure derivation. The signals at δ 4.49, 4.75, 5.25, and 5.30 ppm were assigned to the anomeric protons of the terminal β-d-Gal*p*, (1→3)-linked β-d-Gal*p*, (1→4)-linked α-d-Glc*p*, and (1→4,6)-linked α-d-Glc*p*, respectively. The chemical shifts at δ 3.4 to 4.2 ppm were assigned to the proton signals of carbons C-2 to C-6 of the sugar rings. NMR information was consistent with the FT-IR results, and the possibility of the structure of a few 1,3-linked Gal*p* residues inside the Glc*p* main chain could be excluded.

**Table 2:** ^13^C-NMR and ^1^H-NMR chemical shifts for resonances of glycosyl residues.

Residues	Chemical Shift (δ, ppm)					
	C1/H1	C2/H2	C3/H3	C4/H4	C5/H5	C6/H6
→4)-α-Glc*p*-(1→	99.92/5.25	71.45/3.63	73.30/3.97	76.88/3.63	71.25/3.83	60.50/3.71
→4,6)-α-Glc*p*-(1→	98.70/5.30	72.33/3.52	74.70/3.71	78.45/3.61	71.28/3.63	61.20/3.45
β-Gal*p*-(1→	103.78/4.49	72.30/3.54	74.10/3.63	69.94/4.12	76.70/3.71	61.90/3.73
→3)-β-Gal*p*-(1→	102.39/4.75	71.73/3.83	83.10/3.90	69.90/4.17	75.73/3.73	61.90/3.71

**Figure 5:**
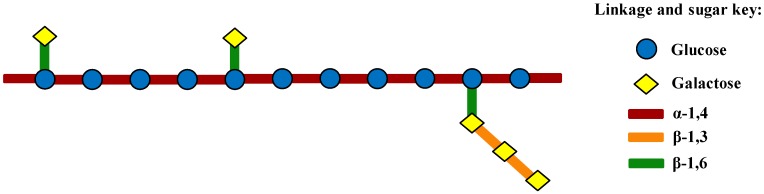
Proposed structural model of MMPX-B2.

Based on the above chemical composition and structural characterizations, the structure of MMPX-B2 might then be inferred and demonstrated as shown in Figure 5. This polysaccharide consisted of (1→4)-linked α-d-Glc*p* residues, partially substituted at C-6 by branches consisting of (1→3)-linked β-d-Gal*p* with terminal β-d-Gal*p* residues.

As the main chain of MMPX-B2 seemed to be composed of (1→4)-linked α-d-Glc*p* residues, which was similar to amylose, a verification experiment was conducted using α-amylase which could cut off the α-(1→4) glycosyl linkage between Glc*p* residues randomly. After being treated with amylase at 40 °C for 5 h, the molecular weight of the MMPX-B2 hydrolysate reduced sharply from 510 kDa to 4.8 kDa. The results verified the deduction that the backbone of MMPX-B2 was composed of (1→4)-linked α-d-Glc*p* residues, which coincided well with the structure derived from the stoichiometric method.

Polysaccharides with β-(1→3) and β-(1→6) glycosyl linkages from clams have rarely been reported. However, a galactan sulfate with a β-(1→3)-glycosidic linkages that was isolated from the marine clam species *Meretrix petechialis* showed anti-HIV activity by inhibiting the syncytia formation [26]. In another experiment, Dai *et al.* obtained a polysaccharide from the clam of *Hyriopsis cumingii* Lea, whose main chain showed to be composed by (1→4)-linked β-d-glucopyranosyl residues, performed immuno-stimulatory activity [27]. Nevertheless, polysaccharides with β-(1→3) and β-(1→6) glycosyl linkages from mushrooms have relatively been deeply studied. Besides, it is known that polysaccharides with β-(1→3) linkages in the main chain and additional β-(1→6) branch points seemed to be necessary for the activity of immunomodulation [28,29]. Thus, considering its structural similarity, MMPX-B2 might possess a similar immuno-regulating function.

### 2.4. Cell-Mediated Immunological Activity

**Figure 6:**
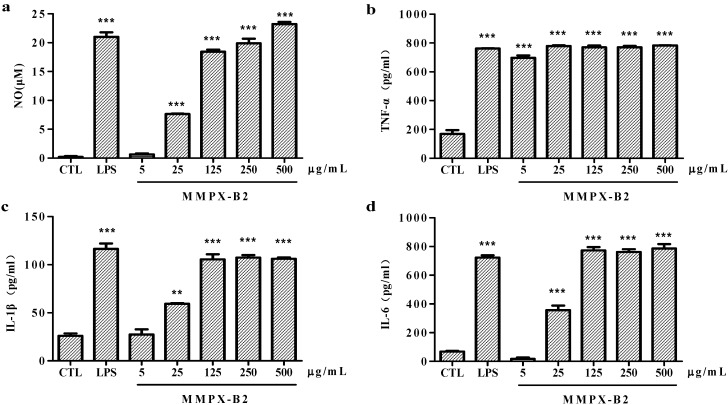
Effects of MMPX-B2 on macrophages-mediated immunity *in vitro*. (**a**) Nitrate accumulation; (**b**) TNF-α; (**c**) IL-1β; (**d**) IL-6. ** is representative of *p* < 0.01 and *** is representative of *p* < 0.001, when compared to the control group.

Cell-mediated immunological tests were then conducted in order to verify the immunological properties of MMPX-B2. The macrophages-mediated immunity plays an important role in the innate immune system. The process of macrophage activation is associated with the production of various inflammatory mediators and cytokines including interleukin (IL), tumor necrosis factor (TNF), and NO [30]. The effects of the immunological activity of MMPX-B2 are indicated in Figure 6. NO, as a messenger or effector, plays important roles in cardiovascular, neural, and immune systems [31]. TNF-α is especially produced by activated macrophages and is involved in systemic inflammation. It is often induced together with IL-6, which plays a major regulatory role in acute local and systemic inflammatory responses [28]. IL-1β is also produced by activated macrophages as a pro-protein and involved in a variety of cellular activities, including cell proliferation, differentiation, and apoptosis [32]. The results showed in Figure 6 indicate that MMPX-B2 possesses immuno-stimulating properties. The levels of NO, IL-6, and IL-1β induced by 125 μg/mL MMPX-B2 matched well with lipopolusaccharide (LPS) and performed in a dose-dependent manner. TNF-α secretion seemed not to be significantly affected by the concentration of the polysaccharide: 25 μg/mL MMPX-B2 already showed comparable effects with 1 μg/mL LPS. Therefore, it seems that there is a correlation between β-(1→3; 1→6) linkages and the immune-regulating effect of MMPX-B2. However, further studies on the immuno-regulation mechanisms are being carried out in order to confirm the initial hypothesis.

## 3. Experimental Section

### 3.1. Materials

The fresh tissue of *M. meretrix* was purchased from Nan Tong Changhua Aquatic Food Co., Ltd (Nantong, Jiangsu, China). Murine monocyte macrophage RAW264.7 was purchased from Cobioer Biosciences Co., Ltd (Nanjing, Jiangsu, China). DEAE-52 cellulose, phosphate buffered saline (PBS), 3-(4,5-dimethyl-2-thiazolyl)-2,5-diphenyl-2H-tetrazolium bromide (MTT), LPS and Griess reagent were purchased from Sigma Chemical Company (St. Louis, MO, USA). The enzymes of trypsin and α-amylase were purchased from Sinopharm Chemical Reagent Co., Ltd (Shanghai, China). Superdex-200 gel prepacked column was purchased from GE Healthcare (Uppsala, Sweden). Enzyme-linked immunosorbent assays (ELISA) kits for measurement of TNF-α, IL-1β, and IL-6, were purchased from Ebioscience Biotechnology Co., Ltd (SanDiego, CA, USA). Penicillin-streptomycin solution (PS) was from Gibco Company (Auckland, Newzealand). Dulbecco's modified eagle medium (DMEM) was picked from Corning Biotechnology Co., Ltd (Tewksbury, MA, USA). Fetal calf serum (FBS) was from PAA Laboratories (Pasching, Austria). The other chemical reagents were of analytical grade and obtained from Sinopharm Chemical Reagent Co., Ltd (Shanghai, China).

### 3.2. Extraction, Isolation and Purification of MMPX-B2

The crude polysaccharide of *M. meretrix* was extracted by modified water extraction method coupled with enzyme hydrolysis. The fresh tissue of *M. meretrix* was firstly homogenized and then extracted with distilled water which was four times the volume of the tissue with additional 2% trypsin (calculated by the wet weight basis of tissue) at 50 °C for 4 h three times [33]. The obtained aqueous extracts were centrifugated at 5000× *g* at room temperature for 10 min. The collected supernatants were concentrated under vacuum at 55 °C. Then, the concentrate was precipitated by 75% ethanol at 4 °C overnight, followed by centrifugation at 5000× *g* for 10 min. The precipitate was collected, dissolved with water and protein impurities were removed with Sevage reagent (chloroform and butanol in the ratio of 3:1) [34]. The mixture was centrifugated and the precipitate was washed twice with absolute ethanol. The crude polysaccharide MMPX was obtained after lyophilization.

MMPX was dissolved in distilled water in order to obtain a solution with the concentration of 5.0 mg/mL. After being loaded onto a DEAE-52 cellulose column (2.6 × 20 cm), MMPX was eluted stepwise with 0, 0.1, and 0.3 M NaCl at a flow rate of 2.0 mL/min. The elutes were collected and lyophilized. Then, the major fraction was further purified by a Superdex 200 dextran column (1.0 × 24 cm) with distilled water as the eluent at a flow rate of 0.3 mL/min. The elutes were collected and lyophilized. The purified polysaccharide MMPX-B2 was then obtained.

### 3.3. Homogeneity and Molecular Weight Determination

The homogeneity and molecular weight of MMPX-B2 were determined by HP-GPC method on a Waters HPLC system (Allances 2695, Waters, Milliford, MA, USA) equipped with a TSK-GEL G3000SWxl column (7.5 × 300 mm) and a refractive index detector (RID) [35]. The PDI was calculated by the ratio of weight-average molecular weight and number-average molecular weight (Mw/Mn). The purified polysaccharide was dissolved in distilled water and eluted with 0.1 M NaCl solution at a flow rate of 0.6 mL/min. The column was calibrated with the Dextran T-series standard samples with different molecular weights (Dextran T 2000, T 500, T 70, T 40, T 10, and T 5).

### 3.4. Analysis of Monosaccharide Composition

GC analysis was employed to detect the monosaccharide composition. MMPX-B2 was hydrolyzed with 2 mol/L trifluoroacetic acid (TFA) (2 mL) at 100 °C for 6 h. The hydrolysate was repeatedly concentrated with methanol until drying, and was then acetylated with 10 mg hydroxylamine hydrochloride and 1 mL pyridine at 90 °C for 30 min. After the addition of acetic anhydride (1 mL), the aldononitrile acetate derivatives were obtained and analyzed by Agilent 6820 GC system (Agilent, Santa Clara, CA, USA) equipped with an OV-17 capillary column (Agilent, USA). The oven was kept at 200 °C for 2 min, then raised to 220 °C at a rate of 15 °C·min^−1^ where it remained for 10 min, and finally increased to 240 °C at a rate of 10 °C·min^−1^ and held for 10 min [36].

### 3.5. FT-IR Analysis

An aliquot of MMPX-B2 was vacuum-dried overnight before being submitted to infrared spectroscopy analysis. FT-IR spectra were recorded on a Nicolet 6700 FT-IR spectrometer (Thermo Scientific, Madison, WI, USA) within the frequency range of 4000–400 cm^−1^ with potassium bromide (KBr) disc method [37].

### 3.6. Periodate Oxidation and Smith Degradation

For the experiment, 20 mg MMPX-B2 was oxidized with 15 mmol/L NaIO_4_ and kept in the dark for nearly 48 h [38]. The absorbance was read at 223 nm every 6 h until the consumption of NaIO_4_ reached a constant value. The reaction was stopped by the addition of 2 mL ethylene glycol. The amount of formic acid generated was titrated with 5 mmol/L NaOH. The solution was dialyzed (Mw cut off: 3000 Da) against distilled water for 48 h and was further reduced by NaBH_4_ for 24 h at 25 °C. The pH value was adjusted to 5.5 with 0.1 mol/L acetic acid. After being hydrolyzed with TFA, the derivatives of alditol acetates were analyzed by GC. The GC method was the same as that of monosaccharide composition analysis.

### 3.7. Methylation Analysis

According to the method of Ciucanu and Kerek, MMPX-B2 was methylated three times using powdered NaOH in DMSO-MeI [39]. The absence of the peak (3700–3200 cm^−1^) corresponding to the hydroxyl group on FT-IR spectra indicated complete methylation. The methylated products were hydrolyzed with 2 mol/L TFA (2 mL) at 100 °C for 6 h and reduced with NaBH_4_. After correcting the pH to 5.5 with 0.1 M acetic acid, the solution was acetylated with equivalent amounts of acetic anhydride pyridine. The resulting products were then examined by GC-MS with a HP-1 capillary column. The following temperature program was employed: 150 °C to 180 °C at a rate of 10 °C/min, then 180 °C to 260 °C at a rate of 15 °C/min and held for 5 min.

### 3.8. NMR Spectroscopy

^13^C-NMR and ^1^H-NMR spectra were obtained on a Bruker DRX-500 NMR spectrometer (Bruker Daltonics, Rheinstetten, Germany). MMPX-B2 was dissolved in D_2_O, and the spectra were recorded at 50 °C. Acetone (not deuterated) was used as the internal standard for the ^13^C analysis.

### 3.9. Hydrolysis of MMPX-B2 with α-Amylase

The polysaccharide of MMPX-B2 was hydrolyzed using α-amylase to verify the backbone structure speculated from chemical characterization. MMPX-B2 was dissolved in distilled water to obtain a solution with the concentration of 1 mg/mL. After the addition of 1% α-amylase (calculated by the weight of MMPX-B2), the enzymatic hydrolysis reaction was carried out at 40 °C for 5 h. The α-amylase in the product was removed using Sevage reagent. The molecular weight of MMPX-B2 hydrolysate was determined with the same method as that of MMPX-B2. The standard samples were Dextran T 40, T 10, T 5, and T 1.

### 3.10. Assay of Cell-Mediated Immunity

#### 3.10.1. Cell Culture

Murine monocyte macrophages RAW264.7 were grown in DMEM medium supplemented with 10% FBS and 1% penicillin-streptomycin (PS) mixed solution under a humidified atmosphere of 5% CO_2_ at 37 °C.

#### 3.10.2. Quantitative Determination of NO and Cytokines

A density of 1.0 × 10^5^ of cells/well were seeded into a 96-well plate and incubated for 16 h at 37 °C. Thereafter, 100 μL of LPS (1 μg/mL, the positive control) or polysaccharide samples (5, 25, 125, 250, 500 μg/mL) were added in, while the equivalent DHEM medium was used as the control. Each group was set with six replicates. The 96-well plate was incubated for 24 h and centrifuged at 1500× *g* for 5 min to collect the supernatant.

To estimate NO level in RAW264.7 cells, nitrite was measured by Griess assay which is a stable reaction product of NO with molecular oxygen [30]. Each cultured supernatant (100 μL) was collected and mixed with 100 μL of Griess reagent. The optical density was measured at 540 nm (SpectraMax M2, Danaher, CA, USA) after 15 min of incubation. The nitrate accumulation was then calculated according to a calibration curve made with a series of known concentrations (2–60 μmol/L) of NaNO_2_ standard solution. The level of TNF-α, IL-1β, and IL-6 were investigated by ELISA kits.

## 4. Conclusions

A water-soluble polysaccharide MMPX-B2 was isolated and purified from the clam of *M. meretrix*. MMPX-B2 showed to be a homogenous polysaccharide, with an apparent molecular weight of 510 kDa. This polysaccharide was composed of residues of d-glucose and d-galactose at a molar ratio of 3.51:1.00. After characterizing the chemical composition and glycosidic linkages, it was hypothesized that MMPX-B2 possessed a main chain of (1→4)-linked α-d-glucopyranosyl residues, partially substituted at the C-6 position with branches consisting of (1→3)-linked β-d-Gal*p* and terminal β-d-Gal*p* residues. The *in vitro* immunological tests with murine macrophage RAW264.7 cells showed that the levels of NO, TNF-α, IL-6, and IL-1β released increased with the addition of MMPX-B2 in a dose-dependent manner, indicating that MMPX-B2 possessed immuno-stimulating properties. The structure-activity relationship was also established.
